# Histone demethylase JMJD1C promotes the polarization of M1 macrophages to prevent glioma by upregulating miR‐302a

**DOI:** 10.1002/ctm2.424

**Published:** 2021-09-26

**Authors:** Chuanhong Zhong, Bei Tao, Feilong Yang, Kaiguo Xia, Xiaobo Yang, Ligang Chen, Tangming Peng, Xiangguo Xia, Xianglong Li, Lilei Peng

**Affiliations:** ^1^ Department of Neurosurgery the Affiliated Hospital of Southwest Medical University Luzhou P. R. China; ^2^ Sichuan Clinical Research Center for Neurosurgery Luzhou P. R. China; ^3^ Department of Rheumatology the Affiliated Hospital of Southwest Medical University Luzhou P. R. China; ^4^ Neurosurgery Department the Affiliated Santai Hospital of North Sichuan Medical College Mianyang 621100 P. R. China

**Keywords:** glioma, histone demethylation, Jumonji domain containing 1C, M1 macrophage polarization, methyltransferase‐like 3, miR‐302a, suppressor of cytokine signaling 2

## Abstract

Glioma is regarded as an aggressive lethal primary brain tumor. Jumonji domain containing 1C (JMJD1C) is a H3K9 demethylase which participates in the progression of various tumors, but its specific function and underlying mechanism in glioma development remain undefined, which is the purpose of our work. We initially assessed JMJD1C expression in glioma tissues and cells using the assays of RT‐qPCR and immunohistochemistry. Meanwhile, the H3K9 level at the microRNA (miR)‐302a promoter region was measured by chromatin immunoprecipitation assay, while luciferase‐based reporter assay was performed for validation of the binding affinity between miR‐302a and methyltransferase‐like 3 (METTL3). The effect of METTL3 on suppressor of cytokine signaling 2 (SOCS2) was subsequently analyzed by MeRIP‐RT‐qPCR. Finally, a xenograft tumor model was established in nude mice, followed by measurement of tumor‐associated macrophages using flow cytometry. JMJD1C was poorly expressed in glioma tissues. Furthermore, JMJD1C increased miR‐302a expression through promoting H3K9me1 demethylation at the miR‐302a promoter region. miR‐302a was identified to target METTL3, which could inhibit SOCS2 expression via m6A modification. JMJD1C promoted M1 macrophage polarization and suppressed the growth of glioma xenografts through the miR‐302a/METTL3/SOCS2 axis both in vivo and in vitro. In conclusion, JMJD1C could enhance M1 macrophage polarization to inhibit the onset of glioma, bringing a new insight into the contribution of JMJD1C to the pathobiology of glioma, with possible implications for targeted therapeutic method.

## Graphical abstract

Our results indicate that JMJD1C enhances M1 macrophage polarization to inhibit the onset of glioma, which brings a new insight for the contributions of JMJD1C to the pathobiology of glioma.

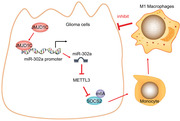

## 1 INTRODUCTION

Glioma, which is the commonest malignancy appearing in human brain, is an essentially incurable disease.^1^ Among all malignant brain tumors, glioma accounts for 81% of cases, which arises in glial cells.^2^ Due to the rapid invasive ability of glioma cells, the mean overall survival of patients with high‐grade glioma is only 15 months.^3^ Thus, it is a matter of urgency to identify new biomarkers for the diagnosis of glioma, which is a challenging goal because of the molecular heterogeneity of the disease.^4^ Accumulating epigenetic analyses have revealed that mutant epigenetic modifiers are common in glioma, and it is likely that dysregulated epigenetic mechanisms participate in the formation of glioma.^5^ DNA methylation and demethylation, as well as histone methylation are involved in epigenetic modification, as may occur in tumor lines.^6^ M1 polarized macrophages are considered to be activated macrophages, which can phagocytose pathogens as part of the innate immune response.^7^ Meanwhile, the activation of macrophages is closely related to the progression of glioma.^8^ In contrast to normal macrophages, tumor‐associated macrophages (TAMs) can exacerbate tumor progression to malignancy by promoting angiogenesis, cell invasion, and migration.^9^

Jumonji domain containing 1C (JMJD1C), which is a family member of lysine demethylase 3,^10^ has the capacity to regulate the aberrant metabolic processes in acute myeloid leukemia.^11^ However, the role of JMJD1C in glioma is rarely discussed. In addition, microRNAs (miRNAs) such as miR‐302c are suggested to participate in modulating the chemosensitivity of glioma cells.^12^ JMJD1C has been shown to bind to the miR‐302 promoter and thus decrease histone H3 lysine 9 (H3K9) methylation.^13^ Interestingly, JMJD1C has been reported to suppress leukemia cell growth by catalyzing H3K9 demethylation.^14^ Another study further notes that miR‐302a is aberrantly expressed in glioma cells and cell lines.^15^ Also, methyltransferase‐like 3 (METTL3) has been found to be upregulated in the tumorigenic glioma stem‐like cells,^16^ while suppressor of cytokine signaling 2 (SOCS2) exerts inhibitory effects on glioma.^17^ Given this background, our study aimed to investigate the functional association between histone demethylase in a series of experiments using glioblastoma multiforme (GBM) cell lines and a nude mouse model xenografted with glioma.

## 2 MATERIALS AND METHODS

### 2.1 Ethics statement

This study was approved by the Ethics committee of the Affiliated Hospital of Southwest Medical University, and signed informed consent had been obtained from participants before the study. Protocols for animal experiment were approved by the Animal Ethics Committee of the Affiliated Hospital of Southwest Medical University.

### 2.2 Bioinformatics analysis

The GEPIA database (http://gepia2.cancer‐pku.cn/#index) was adopted to analyze JMJD1C and SOCS2 expression in glioma samples and normal samples included in the TCGA and GTEx datasets. Then, the binding sites of miR‐302a and METTL3 in the 3′ untranslated region (3′UTR) region was analyzed online (http://www.microrna.org/).

### 2.3 High‐throughput sequencing technology

GBM cell line LN‐229 were treated with plasmids of short hairpin RNA against JMJD1C (sh‐JMJD1C) or negative control (NC) (100 nM; 48 h), followed by extraction of total RNA using TRIzol reagent (15596‐018, Solarbio, Beijing, China). The extraction was repeated three times to collect triplicate samples. The RNA concentration of the samples was identified by a Spectrophotometer (Nanodrop ND‐1000; Thermo Fisher Scientific) set for OD260/280. The sequencing library was generated and sequenced by CapitalBio Technology (Beijing, China), with 5 μg RNA for each sample. rRNA was removed from total RNA using the Ribo‐off^®^ rRNA Depletion Kit (H/M/R) (Vazyme #N406). Next, a library for sequencing was constructed by the VAHTS^®^ Universal V6 RNA‐seq Library Prep Kit for Illumina (NR604), followed by quantification and quality inspection with the KAPA Library Quantitative Kit (KAPA Biosystems). Moreover, the Illumina NextSeqCN500 sequencer was employed for paired‐end sequencing, and sequencing data were saved in the GSE166351 dataset from the GEO database (https://www.ncbi.nlm.nih.gov/geoprofiles/), where differentially expressed genes were identified with the criteria of *p* < 0.05 and fold change > 2. The significantly differentially expressed genes were selected and subject to further experiments. Approximately 41,000 genes and transcripts were tested by the kit, and their expression ratios were obtained by comparing the sh‐JMJD1C and sh‐NC groups. These data were determined by the SBC analysis system (GO and KEGG pathway enrichment analysis). Biological process terminology was implemented by FunRich∖u V3 (http://www.funrich.org/).

### 2.4 Sample source

Tumor and adjacent normal (>2 cm away from tumor tissues) tissues were surgically harvested and confirmed by pathological examinations from 45 glioma patients who received treatment from April 2015 to December 2017 in the Affiliated Hospital of Southwest Medical University. Enrolled patients included 25 males and 20 females aged 19–77 years (52.1 ± 11.5 years). The tissues were pathologically confirmed as specimens of primary glioma. Glioma was graded based on WHO classification (2000), which indicated five cases in grade I, and 10 each in grades II, III, and IV. Patients received no chemo‐ or radio‐therapy before surgery. Portions of the clinical tissue samples were subjected to formaldehyde‐fixation, dehydration, and paraffin‐embedding, which were then put aside for subsequent immunohistochemistry (IHC) and in situ hybridization examinations.

### 2.5 IHC

Paraffin‐embedded clinical tissue specimens were sectioned, dewaxed, dehydrated, and washed with 3% methanol H_2_O_2_. After 10‐min treatment with 10 mM citrate buffer (pH 6.0) at 95°C, the sections were reacted using normal goat serum blocking solution at ambient temperature. Subsequently, the sections were incubated overnight at 4°C using primary antibodies from Abcam (Cambridge, UK) against JMJD1C (ab106457, 1:100) and Ki‐67 (ab15580, 1:200), and were treated with secondary goat anti‐rabbit IgG (ab6785, 1:1000, Abcam) at 37°C on the next day. Following this, the sections were subject to treatment with streptomyces ovalbumin working solution labeled by HRP (0343‐10000U; Immunbio, Beijing, China) at 37°C. Finally, after DAB (Whiga; Guangzhou, China) addition, the sections were then counterstained using hematoxylin, and blued in 1% ammonia water. Following dehydration, clearing, and mounting, 100 cells were observed in each of five randomly selected visual fields under a high power lens.

### 2.6 Cell culture

Glioma cell line LN‐229 and LN‐18 as well as human normal glial cell line HEB were obtained from Tongpai (Shanghai) biotechnology co., LTD (Shanghai, China), while human glioma cell lines U251 cells were from Procell Life Science & Technology Co., Ltd. (Wuhan, Hubei, China). All cells were cultured at 37°C in 5% CO_2_ in the 10% fetal bovine serum (FBS; 10100147; Gibco BRL, Invitrogen, CA, USA)‐contained Dulbecco's modified Eagle medium (DMEM). Besides, HEK293T cells were maintained at 37°C with 5% CO_2_ in the DMEM added with 10% FBS, 100 mg/ml penicillin, and 100 mg/ml streptomycin.

The lentivirus package system consisted of LV5‐GFP (lentivirus‐mediated gene overexpression vector) and pSIH1‐J1‐copGFP (lentivirus shRNA fluorescence expression vector). Plasmids of shRNA targeting JMJD1C (sh‐JMJD1C), shRNA targeting METTL3 (sh‐METTL3), shRNA targeting SOCS2 (sh‐SOCS2) and shRNA against NC (sh‐NC) were synthesized by Gene Pharma (Shanghai, China). Lentivirus package plasmids and the targeted vectors were co‐transfected into HEK293T cells, followed by supernatant collection after 48 h. Virus particles were harvested after the supernatant had been filtered and centrifuged, followed by the detection of virus titer. Cells were identified as sh‐METTL3‐1, sh‐METTL3‐2, sh‐JMJD1C‐1, sh‐JMJD1C‐2, JMJD1C overexpression vector (OE‐JMJD1C), METTL3 overexpression vector (OE‐METTL3), the corresponding NCs (sh‐NC or OE‐NC), OE‐JMJD1C and sh‐NC, OE‐JMJD1C and sh‐SOCS2 or OE‐METTL3 and OE‐SOCS2 according to the transfected plasmids or infected lentivirus. Cells were trypsinized and made into cell suspension, and subsequently cultured for 48 h in a 6‐well plate (5 × 10^4^ cells/ml; 2 ml/well).

### 2.7 Isolation and quantification of RNAs

TRIzol reagents were employed for total RNA extraction, followed by determination of RNA concentration.^18^ Following this, we employed reverse transcription using a one‐step miRNA RT kit (HaiGene Biotech, Harbin, China) and a complementary DNA (cDNA) reverse transcription kit (Reanta, Beijing, China). RT‐qPCR was performed in a fluorescent quantitative PCR instrument, and the relative gene expressions were calculated using 2^−△△^CT method as normalized to the expression of U6 or glyceraldehyde‐3‐phosphate dehydrogenase (GAPDH).^19^ The primers listed in Table S1, Supporting Information, were synthesized by Takara Company (Dalian, China).

### 2.8 Immunoblotting

Total protein extraction was implemented from cells with protease inhibitor‐supplemented RIPA lysis buffer, and the protein concentration was identified with a bicinchoninic acid (BCA) kit. Subsequent to separation, the protein samples were transferred onto polyvinylidene fluoride membranes (Millipore, Billerica, MA, USA). Next, the blocked membranes using 5% bovine serum albumin (BSA) were subsequently incubated with diluted primary antibodies from Abcam (1:500) against JMJD1C (ab106457), METTL3 (ab195352), SOCS2 (ab3692), IgG (ab109489), N6‐methyl‐adenosine (m6A) (ab151230) and H3K9me1 (ab9045) overnight (4°C), followed by 1‐h incubation with the secondary goat anti‐rabbit labeled by HRP (ab205719; 1:2000; Abcam) at ambient temperature. Thereafter, the membrane was added with ECL solution (WBKlS0100, Millipore, MA, USA) for 1 min of incubation and developed by adding VILBER FUSION FX5 developing solution (Vilber Lourmat, France). The protein bands in each group of immunoblotting images were quantified using Image J analysis software (NIH, Washington, DC, USA) with GAPDH as the internal reference.

### 2.9 In situ hybridization^20^

Paraffin‐embedded sections of glioma tissues were deparaffinized and then rehydrated. The activity of endogenous peroxidase was quenched with 3% H_2_O_2_ (30 min). After being detached with proteinase K, the slides were fixed with 4% paraformaldehyde and incubated for 2 h at 60°C in hybridization buffer, followed by overnight incubation with either miR‐302a or a scrambled miR‐302a control probe (50 nM; digitoxin‐labeled LNA probe) also at 60°C. The next day, slides were washed with 2 × SSC with 50% formamide and PBST. The ALP substrates (azocyan tetrazole/5‐bromo‐4‐chloro‐3‐indolyl‐phosphate) were used for the ALP reaction, whereupon slides were mounted with an aqueous sealant and microscopically observed using an Olympus BX‐60 microscope (Olympus, Tokyo, Japan).

### 2.10 EdU assay

We assessed the cell viability with the use of a Cell‐Light EdU DNA Cell Proliferation Kit (RiboBio Co., Ltd, Guangzhou, China). In brief, the cells in 24‐well plate were cultured for 12 h, fixed with 4% formaldehyde, and reacted for 30 min using 1 × Apollo^®^ reaction buffer (100 μl). Next, the cell nuclei were stained using EdU and Hoechst for 30 min, followed by microscopical observation (FM‐600, Puda Optical Instrument, Shanghai, China) with 6–10 fields randomly selected.

### 2.11 CCK‐8 assay

Cells were seeded into 96‐well plates (2 × 10^3^ cells/well), with the culture medium without cells set as the blank control. At 24 h post cell transfection, cell proliferation was assessed with the use of a CCK‐8 kit. The absorbance at 450 nm was then assessed by means of a microplate reader.

### 2.12 Isolation of CD14^+^ peripheral blood mononuclear cells

The whole blood samples were obtained from healthy volunteers for peripheral blood mononuclear cell (PBMC) isolation by means of density gradient centrifugation following the instructions of the Ficoll‐Paque™ Plus kit (Amersham, Little Chalfont, UK). The diluted cellular fraction was covered with Ficoll‐Paque Plus solution, followed by centrifugation (900 × g; 30 min). After twice washing with MACS^®^ Buffer, we isolated CD14^+^ monocytes from the PBMCs by employing CD14 positive magnetic bead‐assisted sorting in the MACS system, as per the manufacturers’ instructions (Miltenyi BiotecGmbH, Bergisch Gladbach, Germany). The purity of CD14^+^ monocytes was consistently higher than 95%, as assessed by flow cytometry.

### 2.13 Co‐culture of CD14^+^ PBMCs with glioma cells

Stably expressed glioma cells (LN‐229 or U251 cells) infected with lentivirus were subjected to seeding into the 0.4‐μm insert (which was permeable to the supernatant, but impermeable to cellular components) of 24‐well Transwell plates (Corning, NY, USA; 1–9 × 10^5^ cells/insert). CD14^+^ monocytes were added to the basolateral chambers and were stimulated with 100 ng/ml of phorbol‐12‐myristic acid‐13‐acetate (PMA; Sigma, St Louis, MO, USA) for induction of macrophage differentiation based on the effector‐to‐target (E/T) ratio (50:1). Cells were subjected to co‐culture and then harvested.

### 2.14 Xenograft tumors in nude mice

Under Specific Pathogen Free conditions, 60 female BALB/c nude mice were housed at 25°C under a 12‐h light/dark cycle. Mice were subcutaneously injected with 2.5 × 10^6^ U251 cells stably infected with OE‐NC, OE‐JMJD1C, OE‐NC and sh‐NC, OE‐JMJD1C and sh‐NC or OE‐JMJD1C and sh‐SOCS2 (*n* = 10 in each group) in 0.1 ml PBS. Tumors were visible one week after injection, and the tumor size was subsequently assessed with Vernier calipers at one‐day intervals. Then, tumor volume was calculated: volume = (long diameter × short diameter^2^)/2. After 10 consecutive daily measurements, mice were euthanized with an intraperitoneal injection of 3% pentobarbital sodium, whereupon the tumor was extracted and used for further experiments.

### 2.15 TAMs and tumor cell isolation

A tumor dissociation kit (Miltenyi BiotecGmbH) was adopted for preparation of single cell suspension from fresh tumors. Cells were promptly isolated by means of a negative magnetic bead‐assisted sorting assay. TAMs were separated from positive cell suspension by means of MagniSort™ Mouse CD11b Positive Selection Kit (Cat. No. 8802‐6860‐74) following the manufacturer's instructions. The schematic image on the separation procedure of tumor cells and TAMs is shown in Figure S2, Supporting Information.

### 2.16 Flow cytometry

Cell suspension was collected, washed and blocked by means of Fc receptor (FcR)‐blocking agent (human/mouse), which was followed by staining with antibodies (BD Biosciences, La Jolla, CA, USA) against CD206‐fluorescein isothiocyanate (FITC) (human), CD206‐PE (mouse), CD86‐PE (human), CD86‐APC.Cy7 (mouse), CD14‐APC (human), or CD11b‐PE (mouse). The mouse macrophages were isolated using Cytofix/CytoPerm Plus™ (BD Biosciences) and then immmunostained with CD206 and CD11b antibodies. A BD Accuri C6 flow cytometer was applied for cell examination, and homologous isotype control antibodies were adopted.

### 2.17 Methylated RNA immunoprecipitation‐qPCR

Confluent LN‐229 cells (1 × 10^7^ cells) were lysed in RIP lysis buffer using the Magna RIP™ Kit (Millipore), followed by collection of the lysate supernatant. Then, the supernatant was separated into two equal parts as inputs. Next, 100 μl cell extract was developed by addition of magnetic beads‐contained RIP buffer (900 μl), which coated with primary mouse antibody against m6A (ab208577, 1:1000, Abcam) or NC mouse IgG and incubated overnight at 4°C. Following rapid centrifugation, the magnetic separator was used, with supernatant discarded. After that, the centrifuge tube was treated using RIP Wash Buffer (500 μl), and the supernatant was again discarded after centrifugation, with six repetitions of the washing step. After the final washing, 50 μl of the mixture centrifuged, and the precipitate was added with the loading buffer to conduct immunoblotting, which served to confirm that the magnetic beads were coated with antibodies. Then remaining samples portions were treated at 55°C with proteinase K for 30 min in a centrifuge tube, with protein detachment by constant shaking. Trizol‐chloroform was then utilized to isolate immunoprecipitated RNA, whereupon RT‐qPCR was utilized for SOCS2 enrichment detection.

### 2.18 RNA pull‐down assay

Biotin‐labeled miR‐302a or simulated NC was transfected into LN‐229 cell line. Streptavidin beads were used to extract biotin‐labeled miRNAs from the cell lysate as per the manufacturer's instructions, followed by incubation, elution, and de‐crosslinking.

### 2.19 Chromatin immunoprecipitation assay

This experiment was conducted using a ChIP kit (Millipore, USA). Upon attaining 70–80% confluence, cells were subjected to formaldehyde‐fixation (final concentration: 1%) at ambient temperature (10 min) to generate DNA‐protein crosslinking. Then, 0.125 M glycine was added to terminate the crosslink, followed by lysis of the cells through ultrasonic treatment. The DNA in cells was broken into 500–1000 bp fragments and the mixture was centrifuged at 13,000 rpm (4°C). The supernatant was separated into three tubes, added with the NC antibody against normal human IgG, the positive control antibody against RNA polymerase II, and rabbit anti‐JMJD1C (1:100, 17‐10262, EMD Millipore, Bedford, Massachusetts, USA) respectively, for overnight incubation at 4°C. The next day, the endogenous DNA‐protein complex was precipitated by addition of ProteinG Dynabeads, followed by the supernatant removal after a brief centrifugation. Thereafter, the nonspecific complex was washed de‐crosslinked overnight at 65°C. Phenol/chloroform addition was adopted for DNA fragment purification and recycling, followed by RT‐qPCR using primers in Table S2, Supporting Information.

### 2.20 Dual‐luciferase reporter gene assay

PmirGLO‐METTL3‐wild type (WT) and PmirGLO‐METTL3‐mutant (MUT) were constructed and then co‐transfected with miR‐302a overexpression plasmids or NC plasmids into 293T cells. Cells were lysed at 48 h after transfection and then harvested. Luminescent signal was detected by means of Dual‐Luciferase^®^ Reporter Assay System. Relative luciferase activity was calculated as relative luciferase activity of firefly luciferase/RLU activity of renilla luciferase.

### 2.21 TUNEL staining

Glioma sections were dewaxed, rehydrated, and then incubated in 1% protease K diluent (50 μl) for 30 min at 37°C. POD activity was eliminated by preincubating with 0.3% H_2_O_2_ methanol solution. The sections were then reacted for 1 h with TUNEL solution in the dark, and further incubated upon addition of 50 μl Converter‐POD. Afterwards, the sections were developed by addition of 2% DAB for 15 min with incubation at ambient temperature followed by microscopical observation (CX43, Olympus). When brownish‐yellow nucleus appeared, the reaction was terminated using distilled water, followed by counterstaining and microscopical observation in 10 randomly chosen fields. The cells presenting with brownish‐yellow nucleus were apoptosis‐positive cells, while the cells with blue nucleus were deemed as normal cells.

### 2.22 Statistical analysis

Statistical analysis for all data in the current study was implemented by SPSS 21.0 software. Measurement data were presented as a form of mean ± standard deviation (SD). Paired *t*‐test was used for data comparison between glioma and adjacent normal tissues. The data conforming to normal distribution and homogeneous variance were subjected to analysis by means of independent sample *t‐*test (for two‐group data) or one‐way ANOVA with Tukey's test (for multi‐group data). Data comparison among groups at varied time points was implemented by means of repeated measures ANOVA with Bonferroni's test. *p* < 0.05 was suggestive of statistically significant.

## 3 RESULTS

### 3.1 JMJD1C is downregulated in glioma and associated with immune response

**FIGURE 1:**
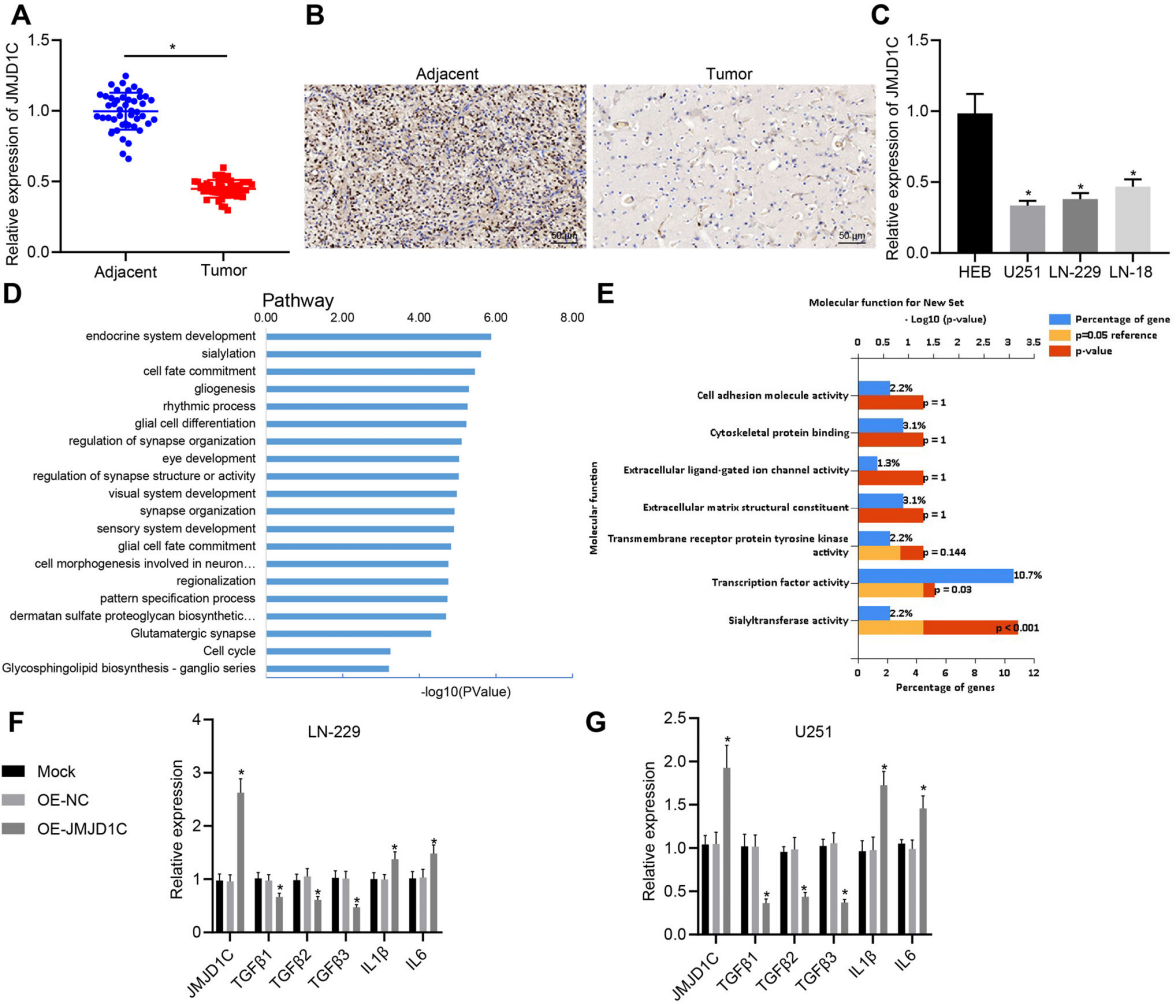
JMJD1C is poorly expressed in glioma and its expression relates to immune response. (A) The expression of JMJD1C in glioma tissues (*n* = 45) and adjacent normal tissues (*n* = 45) detected using RT‐qPCR. * *p* < 0.05 compared with adjacent normal tissues. (B) The expression of JMJD1C in glioma tissues (*n* = 45) and adjacent normal tissues (*n* = 45) determined using IHC (scale bar  =   50 μm). (C) The level of JMJD1C in cell lines HEB, U251, LN‐229, and LN‐18 detected by RT‐qPCR. * *p* < 0.05 compared with cell line HEB. (D) The enrichment of JMJD1C‐associated genes determined with KEGG pathway enrichment analysis. (E) The enrichment of JMJD1C‐related genes in the immune response of biological process related genes. (F) mRNA levels in JMJD1C overexpressed LN‐229 cell line detected by RT‐qPCR (48 h). (G) mRNA levels in JMJD1C overexpressed U251 cell line detected by RT‐qPCR (48 h). * *p* < 0.05 compared with cells treated with OE‐NC. OE‐NC was set as controls. The measurement data are expressed as mean ± SD. Comparison between glioma tissues and adjacent normal tissues is performed using paired *t*‐test, while comparison among multiple groups is conducted using one‐way ANOVA and Tukey's post hoc test. The cellular experiment is carried out at least three times independently

According to the bioinformatics analysis results, analysis of JMJD1C expression in glioma samples and normal samples collected by TCGA and GTEX using the GEPIA database suggested a decline of JMJD1C expression in glioma samples (Figure S1, Supporting Information). As reflected by RT‐qPCR and IHC, there was poor expression of JMJD1C in glioma tumor tissues as compared to adjacent normal tissues (Figure 1A,B). Meanwhile, RT‐qPCR displayed lower levels of JMJD1C in U251, LN‐229, and LN‐18 cells compared with HEB cells (Figure 1C). We adopted high‐throughput sequencing technology to investigate the overall gene expression pattern upon silencing JMJD1C in GBM LN‐229 cell line. Subsequently, silencing of JMJD1C changed the expression of JMJD1C target genes (Figure S3, Supporting Information). As reflected by KEGG pathway enrichment analysis, JMJD1C associated genes were enriched in elements of tumor necrosis factor (TNF) signaling pathways and immune response processes (Figure 1D,E), suggesting possible role of JMJD1C in the immune response of LN‐229 cells.

Subsequently, levels of immune and inflammation‐related genes were detected in LN‐229 and U251 cell lines, which displayed that overexpression of JMJD1C induced a decrease in TGFβ 1–3 expression as well as an increase in interleukin (IL)‐1β and IL‐6 expression in LN‐229 cells (Figure 1F,G). Therefore, we find that JMJD1C is involved in the immune response of GBM.

### 3.2 JMJD1C promotes M1 macrophage polarization and inhibits glioma in vivo

**FIGURE 2:**
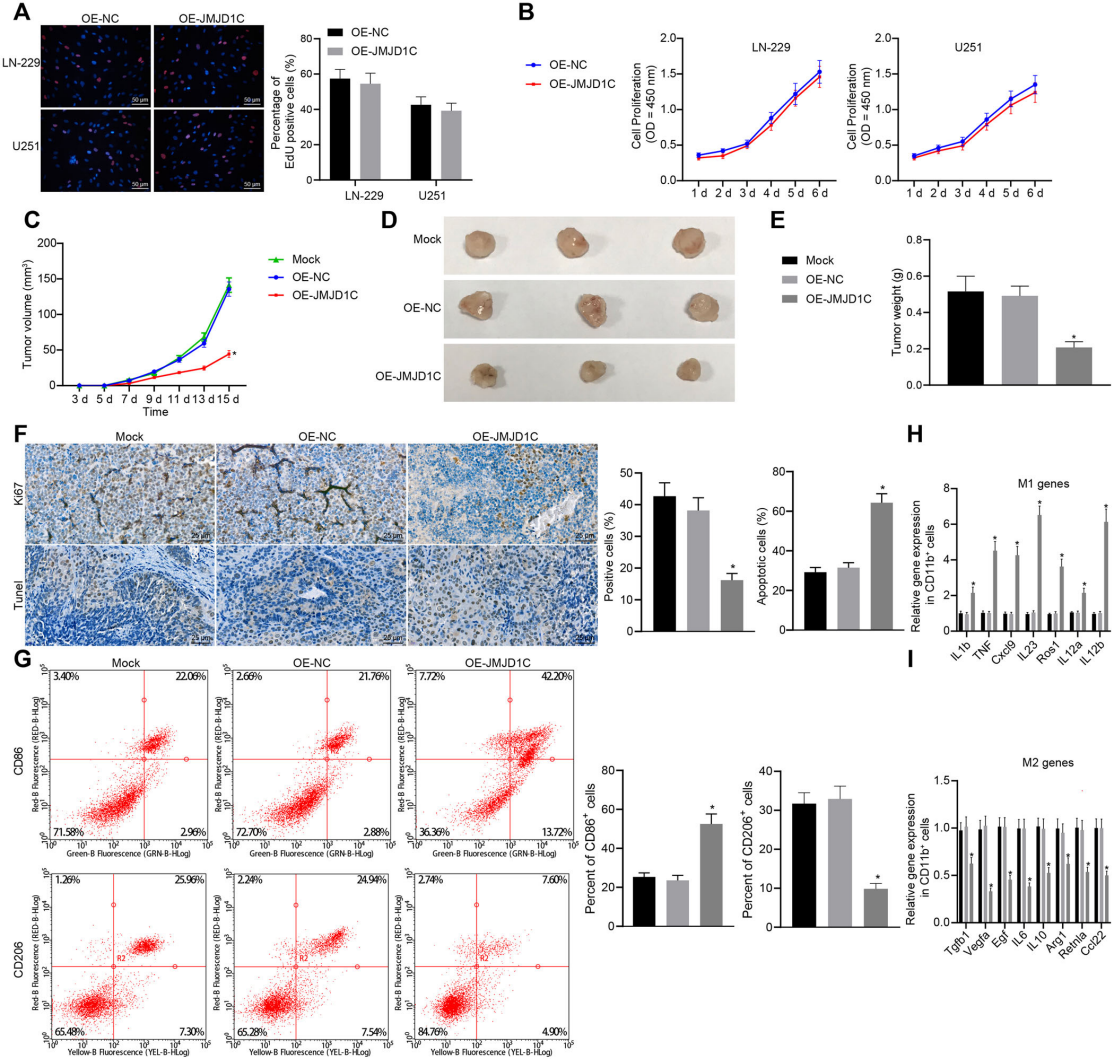
JMJD1C inhibits glioma growth and promotes M1 macrophage polarization in vivo. (A) The proliferation of LN‐229 and U251 cells examined by EdU assay (scale bar  =   50 μm). (B) The proliferation of LN‐229 and U251 cells determined by CCK‐8 assay following stable infection with lentivirus OE‐NC and OE‐JMJD1C. (C) Tumor volume of xenograft tumor mouse models injected with LN‐229 cells infected with OE‐NC or OE‐JMJD1C (*n* = 10). (D), The morphology of BALB/c nude mice tumors injected with LN‐229 cells infected with OE‐NC or OE‐JMJD1C (*n* = 10). (E) Tumor quality of BALB/c nude mice injected with LN‐229 cells infected with OE‐NC or OE‐JMJD1C (*n* = 10). (F) Quantitative analysis for Ki67 positive cells determined by IHC and cell apoptosis assessed by TUNEL staining (scale bar  =   25 μm). (G) The detection of CD206^+^ cells and CD86^+^ cells in isolated CD11b^+^ macrophages by flow cytometry. * *p* < 0.05 compared with the OE‐NC group. (H) Expression of M1 markers in CD11b^+^ cells measured by RT‐qPCR. * *p* < 0.05 compared with the OE‐NC group. (I) Expression of M2 markers in CD11b^+^ cells measured by RT‐qPCR. * *p* < 0.05 compared with the OE‐NC group. The measurement data are expressed as mean ± SD. Comparison between glioma tissues and adjacent normal tissues is performed using paired *t*‐test, while comparison among multiple groups is conducted using one‐way ANOVA and Tukey's post hoc test. Data among groups at different time points are compared using repeated measures ANOVA and Bonferroni's post hoc test. The cellular experiment is carried out at least three times independently

The cell proliferation of LN‐229 and U251 cells stably overexpressing JMJD1C was screened by EdU staining and CCK‐8 assay, which showed that overexpression of JMJD1C exerted functions on glioma cell proliferation in vitro (Figure 2A,B). Next, a mouse model xenografted with tumor was constructed by subcutaneous injection of LN‐229 cells expressing OE‐NC or OE‐JMJD1C. It was observed that overexpression of JMJD1C resulted in inhibition of tumor growth (Figure 2C–E). Results from IHC and TUNEL assays illustrated that overexpression of JMJD1C induced more TUNEL positive cells and fewer Ki67 positive cells compared with treatment of NC overexpression vector (Figure 2F). Mice were euthanized, followed by the extraction of tumor cells and TAM cells (Figure S2, Supporting Information). Then CD86 (M1 macrophage marker) and CD206 (M2 macrophage marker) were determined using flow cytometry, which showed that overexpression of JMJD1C resulted in a decrease of CD206^+^ cells and an increase of CD86^+^ cells in tumor tissues (Figure 2G). Furthermore, RT‐qPCR revealed that that, relative to OE‐NC, M1 markers IL‐1β, TNF, CXCL9, IL‐23, ROS1, IL‐12a, and IL‐12b levels were increased in OE‐JMJD1C treated mice, while expression of M2 markers TGFB1, VEGFA, EGF, IL‐6, IL‐10, ARG1, RETNLA and CCL22 was decreased (Figure 2H,I), suggesting increased M1 macrophage polarization. These results suggest that JMJD1C does not affect glioma cell proliferation per se, but can inhibit glioma cell growth in vivo, which is possibly related to increased M1 macrophage polarization and decreased M2 macrophage polarization.

### 3.3 Specifically expressed JMJD1C accelerates polarization of M1 macrophage

**FIGURE 3:**
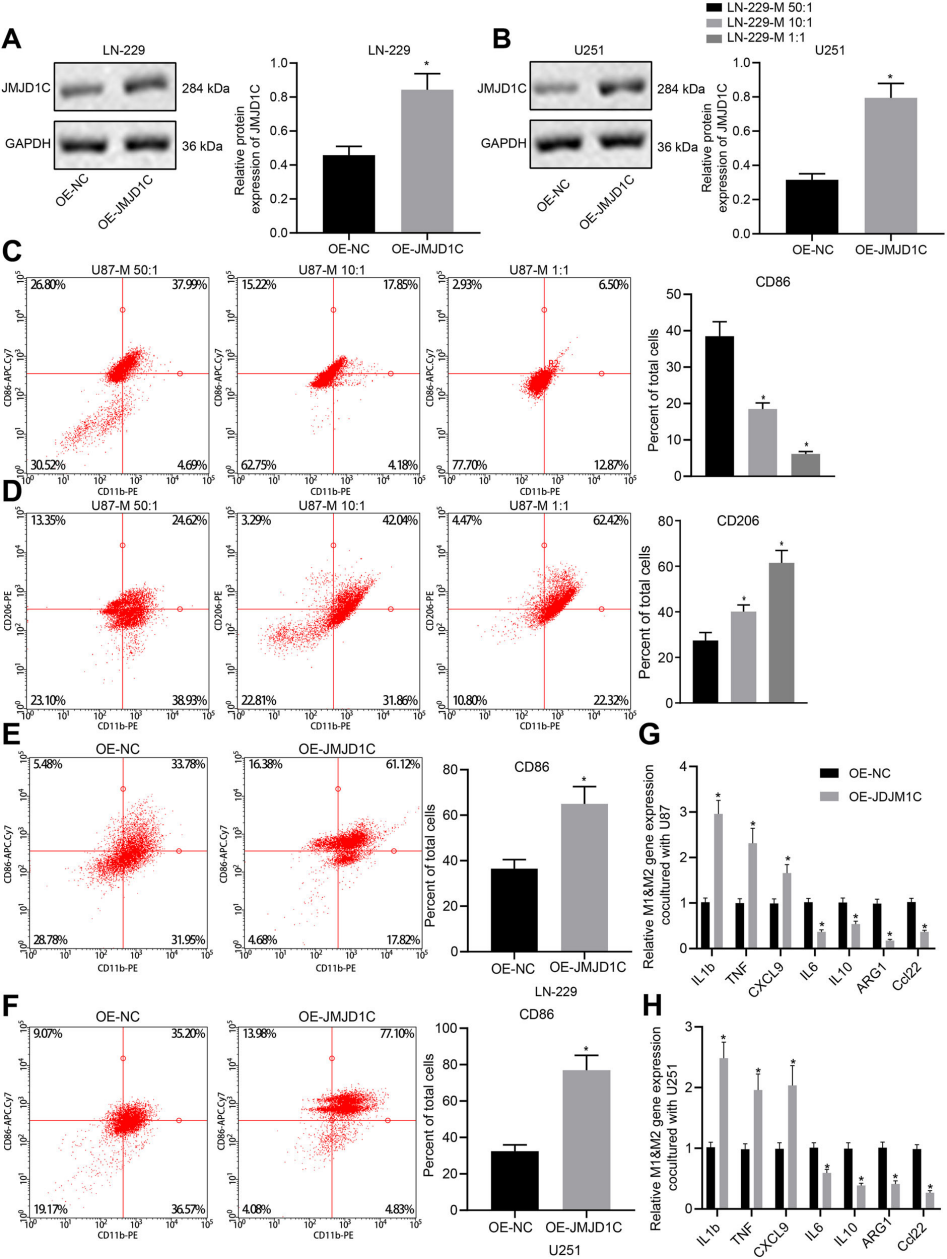
Specific expression of JMJD1C in glioma promotes M1 macrophage polarization in vitro. (A) The protein expression of JMJD1C in LN‐229 cells stably transfected with OE‐NC and OE‐JMJD1C detected by immunoblotting. * *p* < 0.05 compared with the cells treated with OE‐NC. (B) The protein expression of JMJD1C in U251 cells stably transfected with OE‐NC and OE‐JMJD1C detected by immunoblotting. * *p* < 0.05 compared with the cells treated with OE‐NC. (C) Expression of CD86 in human CD14^+^ monocytes co‐cultured with different LN‐229 cells for 4 days detected by quantitative flow cytometry. * *p* < 0.05 compared with the cells treated with LN‐229‐M 50: 1. (D) Expression of CD206 (D) in human CD14^+^ monocytes co‐cultured with different LN‐229 cells for 4 days detected by quantitative flow cytometry. * *p* < 0.05 compared with the cells treated with LN‐229‐M 50: 1. (E) CD14^+^ monocytes co‐cultured with LN‐229 at a ratio of 1:50 for four days, and the expression of CD86 was detected by quantitative flow cytometry. * *p* < 0.05 compared with the cells treated with OE‐NC. (F) CD14^+^ monocytes co‐cultured with U251 at a ratio of 1:50 for four days, and the expression of CD86 was detected by quantitative flow cytometry. * *p* < 0.05 compared with the cells treated with OE‐NC. (G) Expression of macrophage M1 and M2 markers in CD14^+^ monocytes co‐cultured with LN‐229cells measured by RT‐qPCR. * *p* < 0.05 compared with the cells treated with OE‐NC. (H) Expression of macrophage M1 and M2 markers in CD14^+^ monocytes co‐cultured with U251 cells measured by RT‐qPCR. * *p* < 0.05 compared with the cells treated with OE‐NC. The measurement data are expressed as mean ± SD. Comparison between glioma tissues and adjacent normal tissues is performed using paired *t*‐test, while comparison among multiple groups is conducted using one‐way ANOVA and Tukey's post hoc test. The cellular experiment is carried out three times independently

The expression of JMJD1C in LN‐229 and U251 cell lines transfected with OE‐JMJD1C plasmid was tested by immunoblotting (Figure 3A,B). Next, induction of CD86^+^ M1 macrophages that were differentiated from CD14^+^ PBMCs depending on the E/T ratio of effector, with the result that co‐culture with an E/T ratio of 50: 1 showed the best induction effect (Figure 3C,D). Co‐culture assay revealed that JMJD1C elevation increased the number of monocyte‐differentiated CD86^+^ M1 macrophages among LN‐229and U251 cells (Figure 3E,F). In addition, RT‐qPCR results showed that upregulation of JMJD1C promoted the expression of M1 marker genes but inhibited that of M2 marker genes in CD14^+^ PBMCs (Figure 3G,H). Thus, JMJD1C overexpression has the potential to polarize macrophages toward the M1 phenotype.

### 3.4 JMJD1C upregulates miR‐302a by promoting H3K9 demethylation at miR‐302a promoter region

**FIGURE 4:**
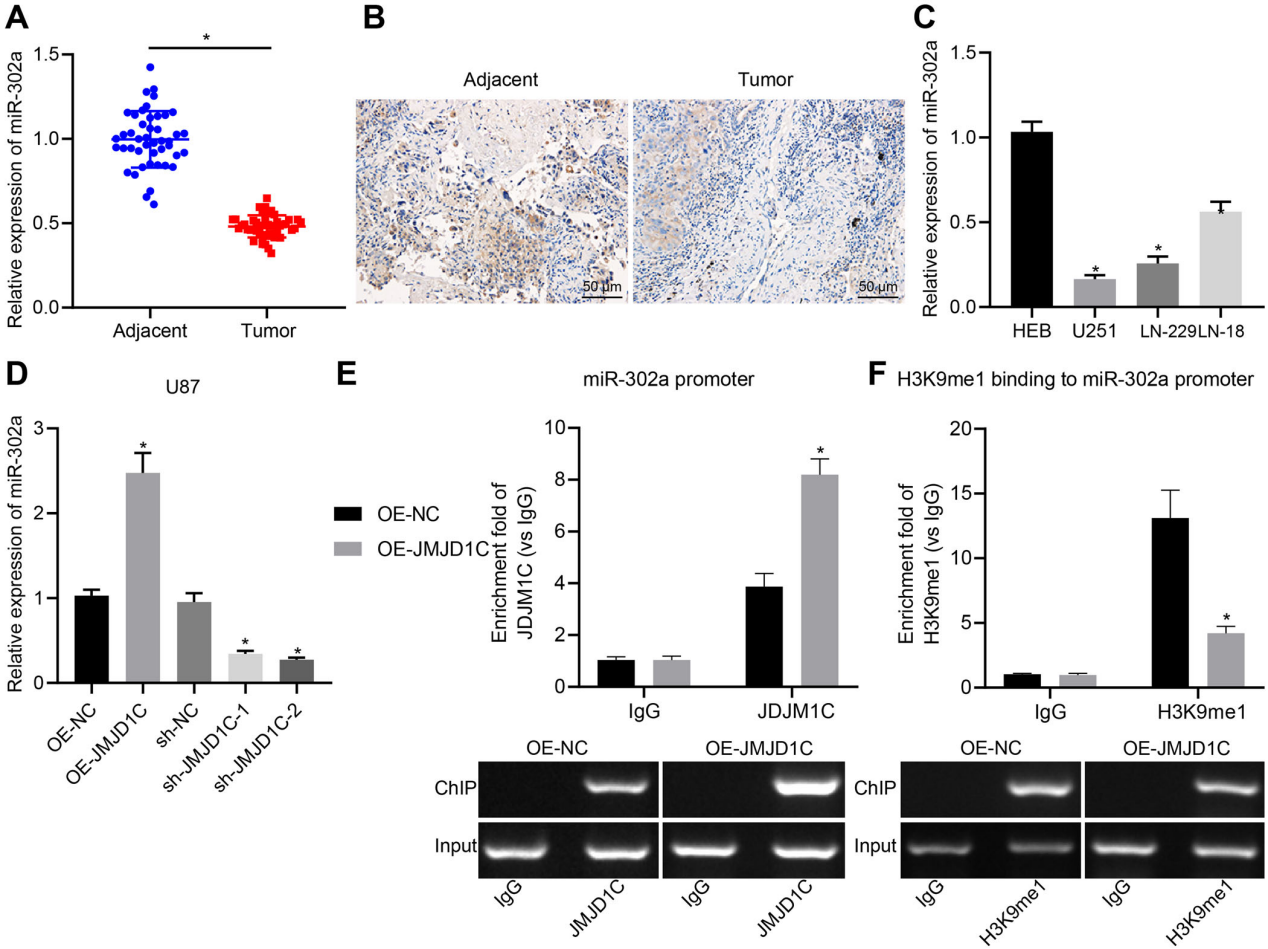
JMJD1C promotes the expression of miR‐302a by regulating H3K9 demethylation. (A) Expression of miR‐302a in glioma tissues (*n* = 45) and adjacent normal tissues (*n* = 45) detected by RT‐qPCR. * *p <* 0.05 compared with adjacent normal tissues. (B) Expression of miR‐302a in glioma tissues (*n* = 45) and adjacent normal tissues (*n* = 45) detected by in situ hybridization (scale bar  =   50 μm). (C) Expression of miR‐302a in cell lines HEB, U251, LN‐229, LN‐18 measured by RT‐qPCR. * *p* < 0.05 compared with human normal glial cell line HEB. (D) Expression of miR‐302a in cell line LN‐229 in response to OE‐JMJD1C or sh‐JMJD1C measured by RT‐qPCR. (E) JMJD1C enrichment in the promoter region of miR‐302a examined using ChIP assay. * *p* < 0.05 compared with cells treated with OE‐NC. (F) H3K9me1 enrichment in the promoter region of miR‐302a examined using ChIP assay. * *p* < 0.05 compared with cells treated with OE‐NC. The measurement data are expressed as mean ± SD. Comparison between glioma tissues and adjacent normal tissues is performed using paired *t*‐test, while comparison among multiple groups is conducted using one‐way ANOVA and Tukey's post hoc test. The cellular experiment is carried out at least three times independently

Based on previous literature, we speculated that JMJD1C may affect glioma cell proliferation by regulating miR‐302a. As expected, results from RT‐qPCR assay and in situ hybridization assay demonstrated that miR‐302a downregulation occurred in glioma tissues (Figure 4A,B). Meanwhile, the expression level of miR‐302a was also reduced in glioma cells, as detected by RT‐qPCR (Figure 4C). However, the level of miR‐302a was increased in JMJD1C overexpressed LN‐229 cells, and was significantly decreased in JMJD1C‐silenced cells (Figure 4D). Then, we found that the enrichment of JMJD1C detected by ChIP assay (Figure 4E) was higher in OE‐JMJD1C treated cells than in OE‐NC treated cells. ChIP results displayed that the level of H3K9me1 was decreased after overexpression of JMJD1C (Figure 4F), showing that JMJD1C may promote the expression of miR‐302a through demethylation of H3K9.

### 3.5 miR‐302a targets METTL3

**FIGURE 5:**
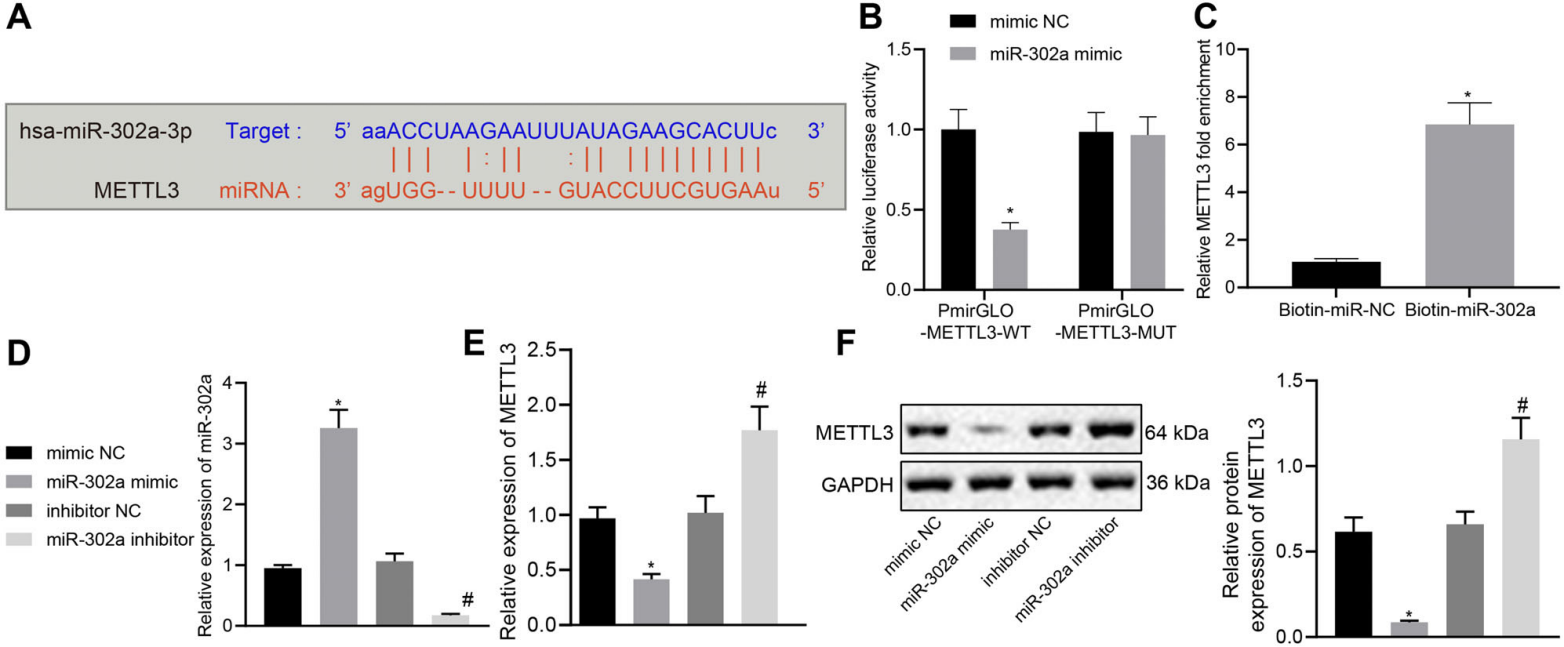
miR‐302a targets METTL3 and negatively regulates its expression. (A) Bioinformatics analysis predicted the targeting relationship between miR‐302a and METTL3. (B) Dual‐luciferase reporter assay validated the relationship between miR‐302a and METTL3. (C) The expression of METTL3 in LN‐229 cells incubated with biotin‐labeled miR‐302a detected by RT‐qPCR; * *p* < 0.05 compared with Biotin‐miR‐NC. (D) The expression of miR‐302a in LN‐229 cells with different treatments detected by RT‐qPCR. (E) The mRNA expression of METTL3 in LN‐229 cells with different treatments detected by RT‐qPCR. * *p* < 0.05 compared with cells treated with mimic NC. # *p* < 0.05 compared with cells treated with inhibitor NC. (F) The protein expression of METTL3 in LN‐229 cells with different treatments detected by immunoblotting. * *p* < 0.05 compared with cells treated with mimic NC. # *p* < 0.05 compared with cells treated with inhibitor NC. The measurement data are expressed as mean ± SD. Comparison between glioma tissues and adjacent normal tissues is performed using paired *t*‐test. The cellular experiment is carried out three times independently

As predicted by the bioinformatics website, a targeted binding site between miR‐302a and METTL3 3′UTR was identified (Figure 5A). Results of dual‐luciferase reporter assay validated targeting relationship. To be more specific, miR‐302a mimic and PmirGLO‐METTL3‐WT co‐transfection decreased the luminescent signal in the cells, while no such difference was seen in the cells co‐transfected with miR‐302a mimic and PmirGLO‐METTL3‐MUT (Figure 5B). The interaction between miR‐302a and METTL3 was further confirmed by RNA pull‐down assay using biotin‐labeled miR‐302a probes (Figure 5C). The expression change of METTL3 after overexpression or depletion of miR‐302a in LN‐229 cells was determined with RT‐qPCR along with immunoblotting (Figure 5E,F). Efficiency of overexpression and depletion was verified by RT‐qPCR (Figure 5D). Expression of METTL3 was found decreased in the miR‐302a mimic‐treated cells, while it was increased in the miR‐302a inhibitor‐treated cells. These results concur in showing that miR‐302a targets METTL3.

### 3.6 METTL3 inhibits M1 macrophage polarization through degradation of SOCS2 mRNA induced by m6A modification

**FIGURE 6:**
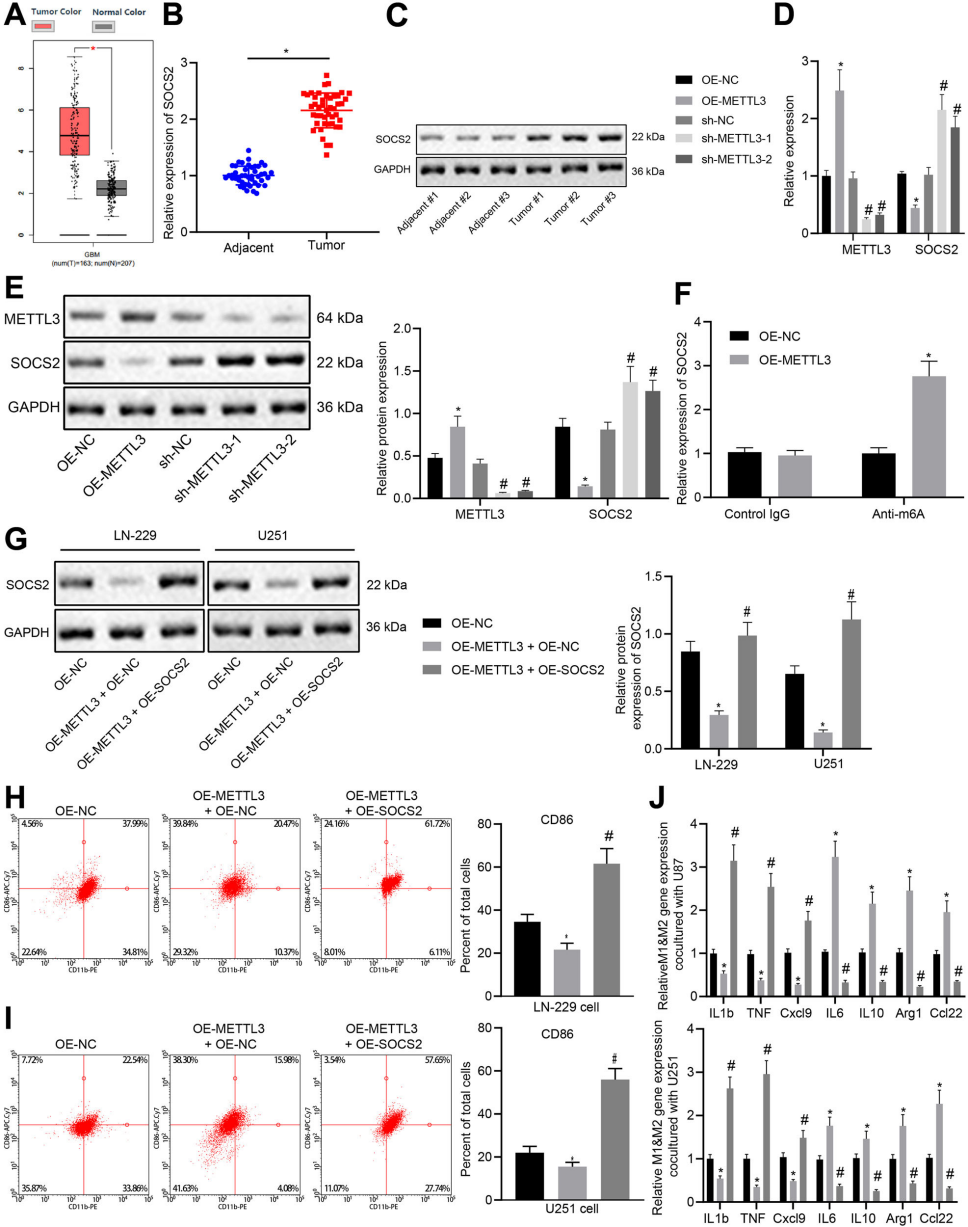
METTL3 promotes M1 polarization of macrophage by promoting the degradation of SOCS2 through m6A modification of SOCS2 mRNA. (A) The relationship between SOCS2 expression and tumor analyzed using GEPIA database. (B) The mRNA level of SOCS2 in glioma tissues (*n* = 45) and adjacent normal tissues (*n* = 45) measured by RT‐qPCR. * *p* < 0.05 compared with adjacent normal tissues. (C) The protein level of SOCS2 in three pairs of glioma tissues and adjacent normal tissues measured by immunoblotting. (D) The mRNA expression of METTL3 and SOCS2 in differently treated cells detected by RT‐qPCR. * *p* < 0.05 compared with cells treated with OE‐NC. # *p* < 0.05 compared with cells treated with sh‐NC. (E) The protein expression of METTL3 and SOCS2 in differently treated cells detected by immunoblotting. * *p* < 0.05 compared with cells treated with OE‐NC. # *p* < 0.05 compared with cells treated with sh‐NC. (F) The methylation level of SOCS2 in glioma cell LN‐229 after overexpression of METTL3 detected by MeRIP‐qPCR. (G) The protein level of SOCS2 in LN‐229 and U251 cells measured by immunoblotting. * *p* < 0.05 compared with cells treated with OE‐NC. # *p* < 0.05 compared with cells treated with OE‐METTL3 and OE‐NC. (H) The quantitative analysis for expression of CD86 detected by flow cytometry after CD14^+^ monocytes were co‐cultured with LN‐229 cells at a ratio of 1:50 for 4 days. * *p* < 0.05 compared with cells treated with OE‐NC. # *p* < 0.05 compared with cells treated with OE‐METTL3 and OE‐NC. (I) The quantitative analysis for expression of CD86 detected by flow cytometry after CD14^+^ monocytes were co‐cultured with U251 cells at a ratio of 1:50 for four days. * *p* < 0.05 compared with cells treated with OE‐NC. # *p* < 0.05 compared with cells treated with OE‐METTL3 and OE‐NC. (J) Expression of macrophage M1 and M2 markers in CD14^+^ monocytes co‐cultured with LN‐229 or U251 cells detected by RT‐qPCR. * *p* < 0.05 compared with cells treated with OE‐NC. # *p* < 0.05 compared with cells treated with OE‐METTL3 and OE‐NC. The measurement data are expressed as mean ± SD. Comparison between glioma tissues and adjacent normal tissues is performed using paired *t*‐test, while comparison among multiple groups is conducted using one‐way ANOVA and Tukey's post hoc test. The cellular experiment is carried out at least three times independently

Furthermore, we investigated the effect of mRNA methylase METTL3 on SOCS2 expression in glioma cells. There was an elevation of SOCS2 in GBM tissues relative to normal tissues, according to the GEPIA database (Figure 6A). The mRNA level of SOCS2 in clinical samples detected by RT‐qPCR exhibited an ascending trend in glioma tissues (Figure 6B). The protein expression of SOCS2 was also increased in glioma tissues (Figure 6C). Subsequently, METTL3 was overexpressed or silenced in LN‐229 cells, which was found to inhibit the SOCS2 mRNA and protein expression, whereas silencing of METTL3 provoked opposite results (Figure 6D,E). Considering that METTL3 is a methyltransferase, the m6A level of SOCS2 in METTL3 overexpressed LN‐229 cells was analyzed using MeRIP‐RT‐qPCR, which displayed that methylation level of SOCS2 increased when METTL3 was overexpressed (Figure 6F). The above results indicate that METTL3 promotes SOCS2 degradation in glioma by promoting m6A methylation modification of SOCS2.

METTL3 was overexpressed or METTL3 and SOCS2 were simultaneously overexpressed in LN‐229 and U251 cells, followed by detection METTL3 and SOCS2 protein expression in differently treated cells. Results revealed that METTL3 overexpression led to an inhibited SOCS2 expression, while co‐transfection of METTL3 and SOCS2 promoted the expression of SOCS2 (Figure 6G). Co‐culture of human CD14^+^ monocytes and glioma cells (LN‐229 or U251 cells) revealed that overexpression of METTL3 induced a decline in the number of CD86^+^ M1 macrophages that had differentiated from monocytes, while simultaneous overexpression of METTL3 and SOCS2 increased the number of differentiated CD86^+^ M1 macrophages (Figure 6H,I). In addition, overexpression of METTL3 resulted in an inhibited expression of M1 markers and an elevated expression of M2 markers, while co‐transfection of METTL3 and SOCS2 promoted the expression of M1 marker genes and inhibited expression of M2 marker genes (Figure 6J). The above data indicate that METTL3 may promote M1 macrophage polarization by inducing degradation of SOCS2 mRNA.

### 3.7 JMJD1C prevents glioma via M1 macrophage polarization induced by miR‐302a/METTL3/SOCS2

**FIGURE 7:**
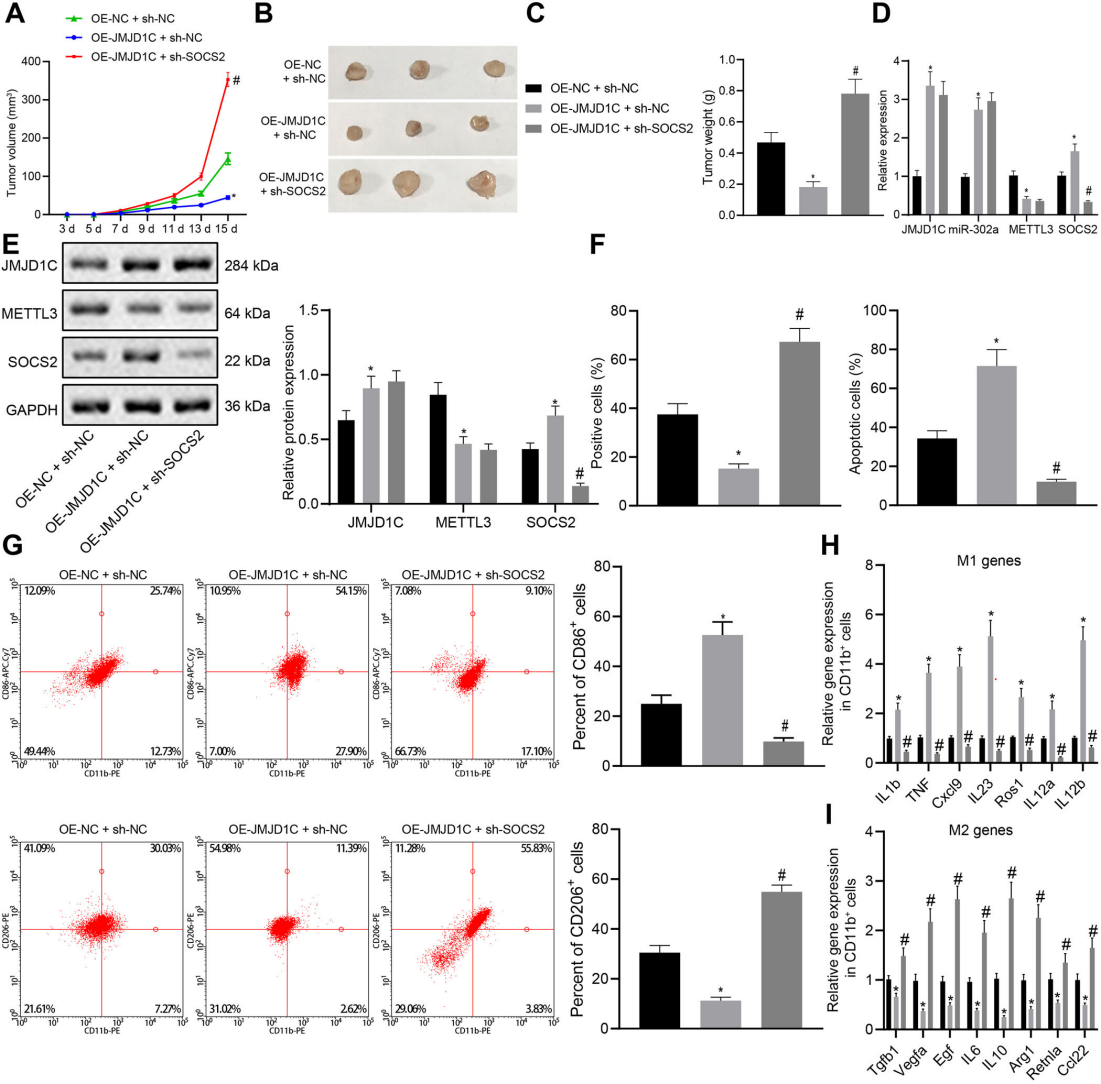
JMJD1C promotes M1 macrophage polarization and inhibits glioma development through miR‐302a/METTL3/SOCS2. (A) Tumor volume of xenograft tumors in mice model (*n* = 10) subcutaneously injected with LN‐229 cells. (B) The morphology of BALB/c nude mice tumors injected with LN‐229 cells (*n* = 10). (C) Tumor quality of BALB/c nude mice injected with LN‐229 cells (*n* = 10). (D) mRNA levels of JMJD1C, miR‐302a, METTL3 and SOCS2 in xenograft tumors of each group. (E) Protein levels of JMJD1C, METTL3, and SOCS2 in xenograft tumors of each group. (F) Quantitative analysis for Ki67 positive cell expression detected by IHC and TUNEL detection on cell apoptosis. (G) Flow cytometry separation of CD11b^+^ macrophages and detection of CD206+ and CD86+ in CD11b^+^ cells. (H) M1 marker gene expression in CD11b^+^ cells evaluated using RT‐qPCR. * *p* < 0.05 compared with cells treated with OE‐NC. # *p* < 0.05 compared with cells treated with OE‐JMJD1C and sh‐NC. (I) M2 marker gene expression in CD11b^+^ cells evaluated using RT‐qPCR. * *p* < 0.05 compared with cells treated with OE‐NC. # *p* < 0.05 compared with cells treated with OE‐JMJD1C and sh‐NC. The measurement data are expressed as mean ± SD. Comparison among multiple groups is conducted using one‐way ANOVA and Tukey's post hoc test. Data among groups at different time points are compared using repeated measures ANOVA and Bonferroni's post hoc test. The cellular experiment is carried out at least three times independently

Finally, to verify whether JMJD1C promotes the polarization of M1 macrophage to inhibit glioma development through the miR‐302a/METTL3/METTL3/SOCS2 axis, we obtained LN‐229 cells stably overexpressing JMJD1C alone or in conjunction with silencing of SOCS2, which were then inoculated to the nude mice. Overexpression of JMJD1C inhibited tumor growth, but this effect was reversed by the addition of sh‐SOCS2 treatment (Figure 7A–C). The expression of JMJD1C, miR‐302a and SOCS2 was increased, while the METTL3 level reduced after OE‐JMJD1C treatment, whereas the expression of JMJD1C, miR‐302a and METTL3 did not statistically change, despite a reduced SOCS2 level in response to OE‐JMJD1C and sh‐SOCS2 (Figure 7D,E).

IHC and TUNEL assays revealed more TUNEL‐positive cells and fewer Ki67‐positive cells after OE‐JMJD1C treatment, whereas the trends were reversed by the further treatment of sh‐SOCS2 (Figure 7F). The results of flow cytometry displayed that the percentage of CD206^+^ cells was decreased and that of CD86^+^ cells was increased in the OE‐JMJD1C, while further treatment of sh‐SOCS2 reversed the results (Figure 7G). Meanwhile, the IL‐1β, TNF, CXCL9, IL‐23, ROS1, IL‐12a and IL‐12b were upregulated in the OE‐JMJD1C, while TGFB1, VEGFA, EGF, IL‐6, IL‐10, ARG1, RETNLA, and CCL22 were downregulated, which the trends were abrogated by the further treatment of sh‐SOCS2 (Figure 7H,I). In conclusion, JMJD1C promotes M1 macrophage polarization and inhibits glioma development through the miR‐302a/METTL3/SOCS2 axis.

## 4 DISCUSSION

M1 polarized macrophages are activated macrophages, which can phagocytose pathogens.^7^ Meanwhile, the activation of macrophages is closely related to the progression of glioma.^8^ However, there has been limited research on the effects of M1 macrophage polarization on glioma development. In this study we report that interruption of histone demethylation or miRNA‐mediated macrophage polarization affected the tumor growth rate in glioma‐implanted mice.

First, the expression profiles of JMJD1C in glioma tumor tissues and cell lines were identified. Dysregulated JMJD1C has been found previously in different diseases such as leukemia and esophageal cancer.^21^, ^22^ However, the detailed mechanism underlying JMJD1C still remains largely unknown. The evidence collected in this study suggest that JMJD1C expression is decreased in glioma, to an extent correlating with the immune response. JMJD1C has also been reported to be associated with immune‐related diseases as a transcriptional factor regulating lineage specification during hematopoiesis.^23^ In GBM cells, overexpression of JMJD1C reduced TGFβ 1‐3 and IL‐10 expression as well as increasing IL‐1β and IL‐6 expression. TGFβ 1–3 and IL‐10 are considered as anti‐inflammatory cytokines, while IL‐1β and IL‐6 are pro‐inflammatory factors.^24^, ^25^ Thus, we can infer that JMJD1C was also involved in the inflammatory response in the glioma model.

Our findings subsequently revealed that JMJD1C promoted M1 macrophage polarization to inhibit glioma progression both in vivo and in vitro. Macrophage polarization is defined as the functional heterogeneity of macrophage phenotypes, in which M1 macrophages inhibit glioma cell proliferation, while M2 macrophages tend to promote proliferation of glioma cells.^26^ M1 macrophages secrete cytokines capable of inhibiting the proliferative capacity of surrounding cells and damaging supporting tissues, where M2 macrophages secrete other cytokines capable of potentiating the proliferative capacity of contiguous cells and tissue repair. Besides, polarization of M1‐M2 macrophages is considered as a strictly controlled process involving an array of pathways for transcriptional and post‐transcriptional regulatory signaling.^27^ The presence of a pool of M2 macrophages has been confirmed in high‐grade gliomas, and predominance of M2‐type macrophages in glioma can induce local and systemic immune suppression.^28^ By contrast, patients with more abundant M1 macrophages have higher overall survival and progression free survival.^29^ Chlorogenic acid (CHA)‐encapsulated mannosylated liposomes promote proliferative capacity of the M2 pro‐tumorigenic phenotype to the favorable M1 anti‐tumorigenic phenotype, thus contributing to inhibited glioma tumor growth.^30^ The data shown in our study indicate that overexpressed JMJD1C increases the expression of M1 markers (IL‐1β, TNF, CXCL9, IL‐23, ROS1, IL‐12a, and IL‐12b) both in the glioma mouse models and in the CD14^+^ monocytes co‐cultured with glioma cells. Therefore, we suppose that JMJD1C may be an essential controller of macrophage polarization serving to regulate inflammatory responses targeting the tumor.

Moreover, we also uncovered that the histone demethylase JMJD1C regulated the expression of miR‐302a. The expression of miRNAs such as miR‐127‐3p can be regulated by histone deacetylase inhibition or DNA demethylation in glioma cells.^31^ Furthermore, JMJD1C could partially control the expression of miRNAs.^32^ Increased miR‐302‐3p expression has been identified in glioblastoma cells, which is related to the cell viability.^33^ Also, the miR‐302‐367 cluster could be a potential therapy for glioblastoma.^34^ Meanwhile, Jumonji C‐domain harboring histone demethylase JMJD1C could demethylate histone H3K9 mono‐ and di‐methylation to modulate transcriptional activation.^35^ Furthermore, JMJD1C suppresses leukemia cell growth by catalyzing H3K9 demethylation.^14^ Next, we found that miR‐302a negatively regulated METTL3 expression. METTL3 has been shown previously to be essential for survival of glioma stem‐like cells.^36^ METTL3 can also catalyze mRNA modification through mA to regulate RNA metabolism,^37^ whereas Knockdown of METTL3 has been demonstrated to abolish SOCS2 mRNA m6A modification.^38^ Furthermore, SOCS are key regulators of immune responses and T helper cell polarization; the M1‐like population was found to be increased in mice with SOCS2 deficiency, and SOCS2 is known to control polarization and plasticity of macrophages by modulating and polarizing stimuli signaling through STATs.^39^ Moreover, SOCS2 can promote M1 macrophage polarization in association with the underlying downstream mechanism.^26^ In view of the importance of SOCS2 in modulating macrophage polarization, we propose that targeting SOCS2 may represent an innovative approache to treat glioma.

## 5 CONCLUSIONS

**FIGURE 8:**
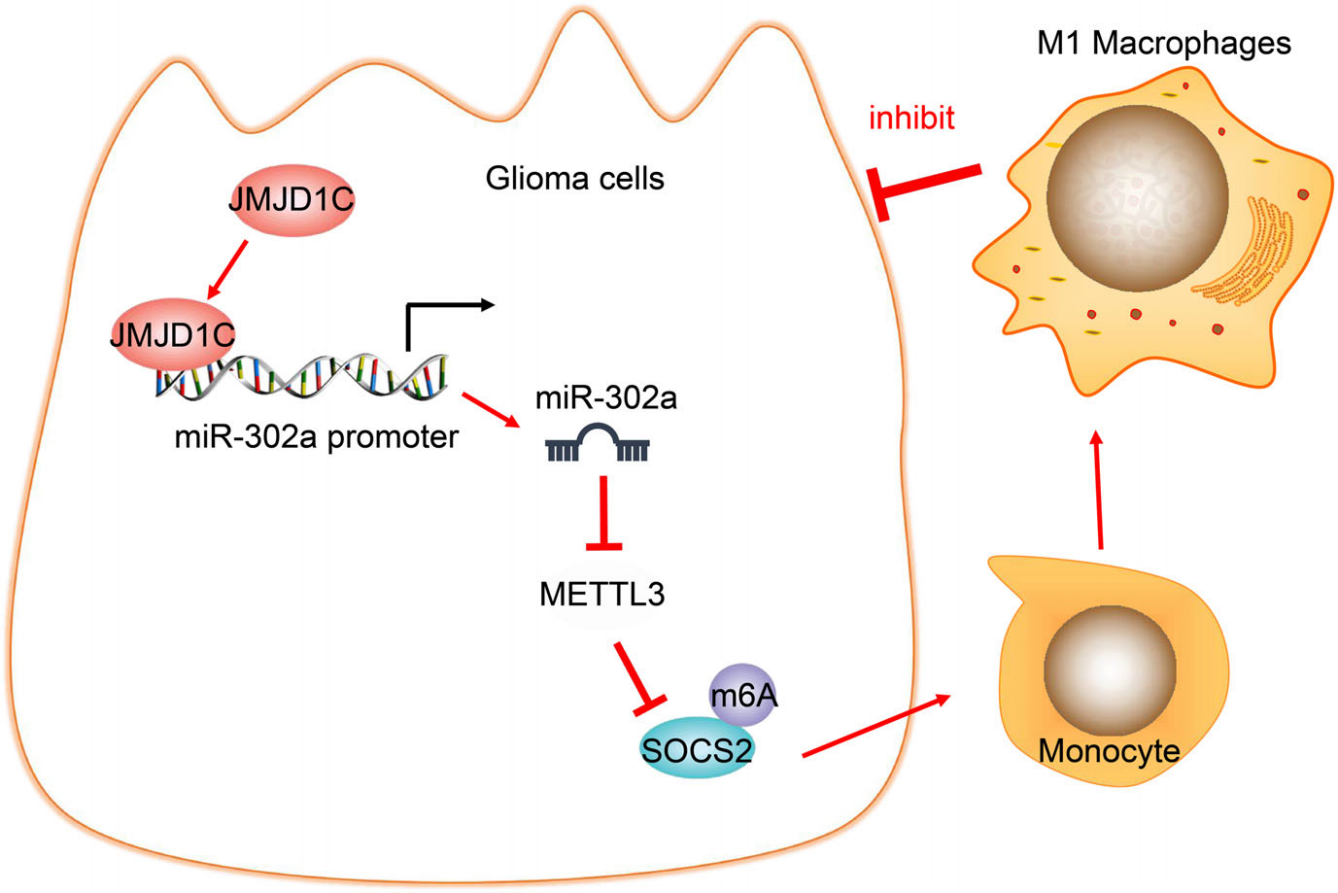
The methylase JMJD1C promotes the expression of miR‐302a through epigenetic modification, inhibits METTL3/SOCS2 and facilitates the polarization of M1 macrophages, thus inhibiting the occurrence of glioma

The role of histone demethylase JMJD1C in glioma has not been comprehensively investigated in previous work. However, the current study provides evidence that JMJD1C plays a tumor‐inhibiting role in glioma by upregulating miR‐302a and further affecting the METTL3/SOCS2‐mediated polarization of M1 macrophages (Figure 8). Nevertheless, future studies are warranted to further explore the possibilities of using JMJD1C as a biomarker for glioma prognosis and treatment.

## ETHICAL APPROVAL AND CONSENT TO PARTICIPATE

This study was approved by the Ethics committee of the Affiliated Hospital of Southwest Medical University, and the signed informed consent had been obtained from participants before study. Protocols for animal experiment were ratified by the Animal Ethics Committee of the Affiliated Hospital of Southwest Medical University.

## AVAILABILITY OF DATA AND MATERIAL

The data that support the findings of this study are available from the corresponding author upon reasonable request.

## CONFLICT OF INTEREST

The authors declare that they have no competing interests.

## AUTHOR CONTRIBUTIONS

Chuanhong Zhong and Bei Tao conceived and designed research. Kaiguo Xia and Xiaobo Yang performed experiments. Lilei Peng analyzed data. Tangming Peng interpreted results of experiments. Xiangguo Xia prepared figures. Xianglong Li drafted manuscript. Ligang Chen edited and revised manuscript. Chuanhong Zhong, Bei Tao, Kaiguo Xia, Xiaobo Yang, Lilei Peng, Tangming Peng, Xiangguo Xia, Xianglong Li, and Ligang Chen approved final version of manuscript.

## Supporting information

**SUPPLEMENTARY FIGURE 1** Analysis on the JMJD1C expression in glioma samples and adjacent normal samples collected by the TCGA and GTEX using the GEPIA database. The abscissa represents the sample type, and the ordinate represents the expression value; the red box diagram represents the tumor sample, and the gray box diagram represents the normal sample.

**SUPPLEMENTARY FIGURE 2** Schematic picture on the separation procedure of tumor cells and TAM

**SUPPLEMENTARY FIGURE 3** Heatmap of some specific JMJD1C target genes

tableS1‐S2
