# Supplementary figures and images for: Histone demethylase JMJD1C promotes the polarization of M1 macrophages to prevent glioma by upregulating miR‐302a
Source: Clin Transl Med. 2021 Sep 26;11(9):e424. doi: 10.1002/ctm2.424 (PMC8473479; doi:10.1002/ctm2.424)

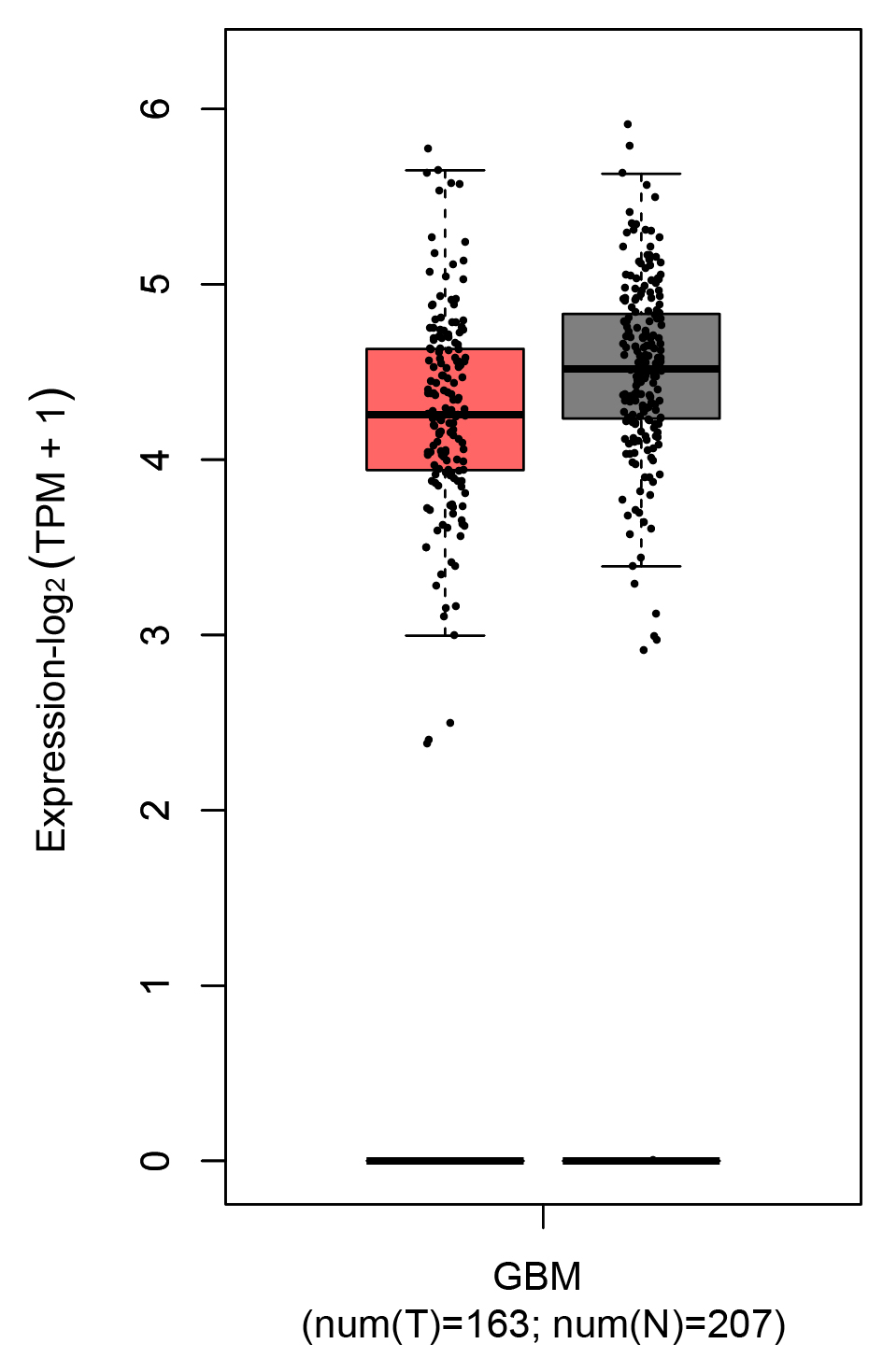

Supplement: Supplementary file 1 — SUPPLEMENTARY FIGURE 1 Analysis on the JMJD1C expression in glioma samples and adjacent normal samples collected by the TCGA and GTEX using the GEPIA database. The abscissa represents the sample type, and the ordinate represents the expression value; the red box diagram represents the tumor sample, and the gray box diagram represents the normal sample. [file CTM2-11-e424-s002.jpg]

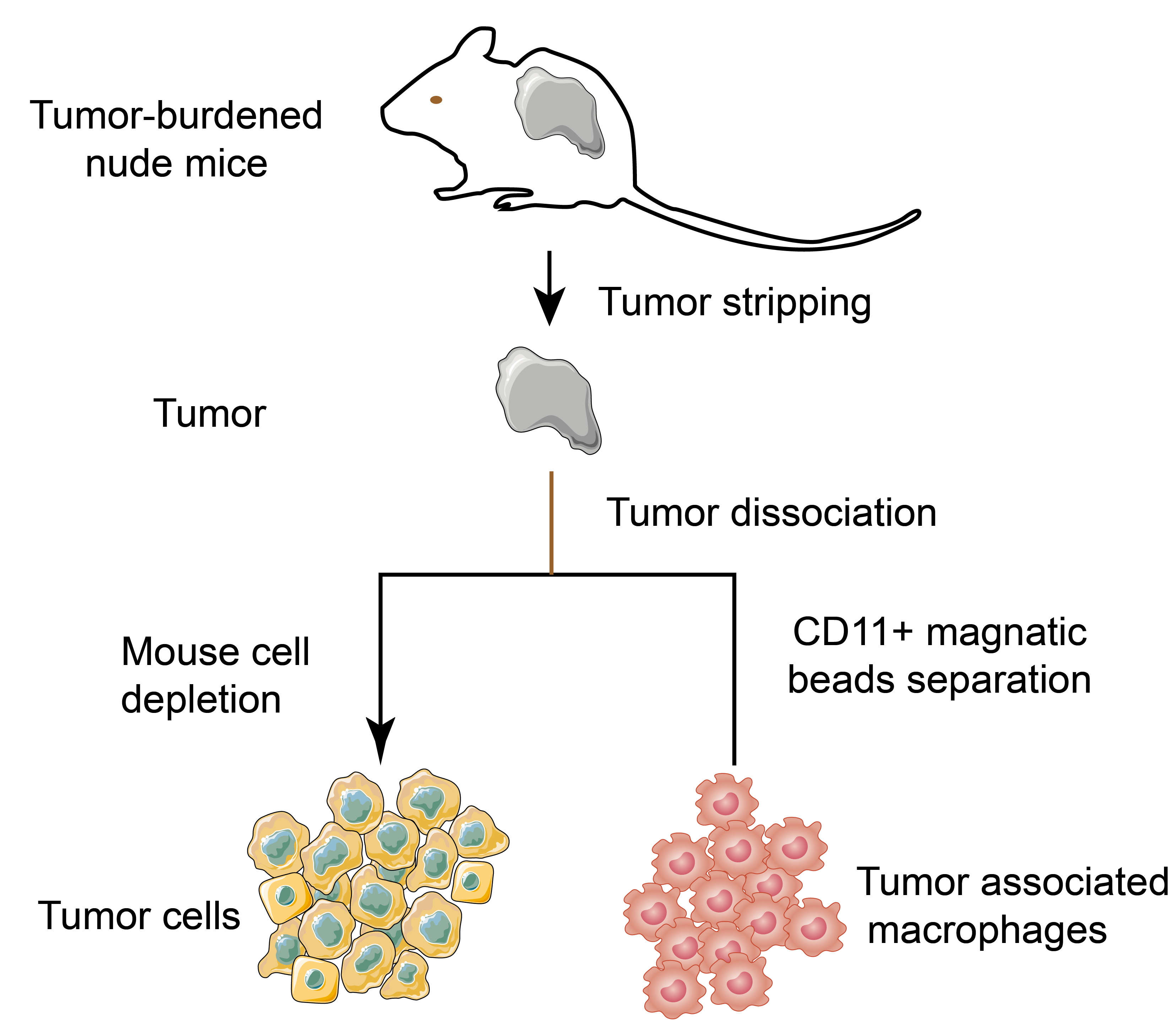

Supplement: Supplementary file 2 — SUPPLEMENTARY FIGURE 2 Schematic picture on the separation procedure of tumor cells and TAM [file CTM2-11-e424-s001.jpg]

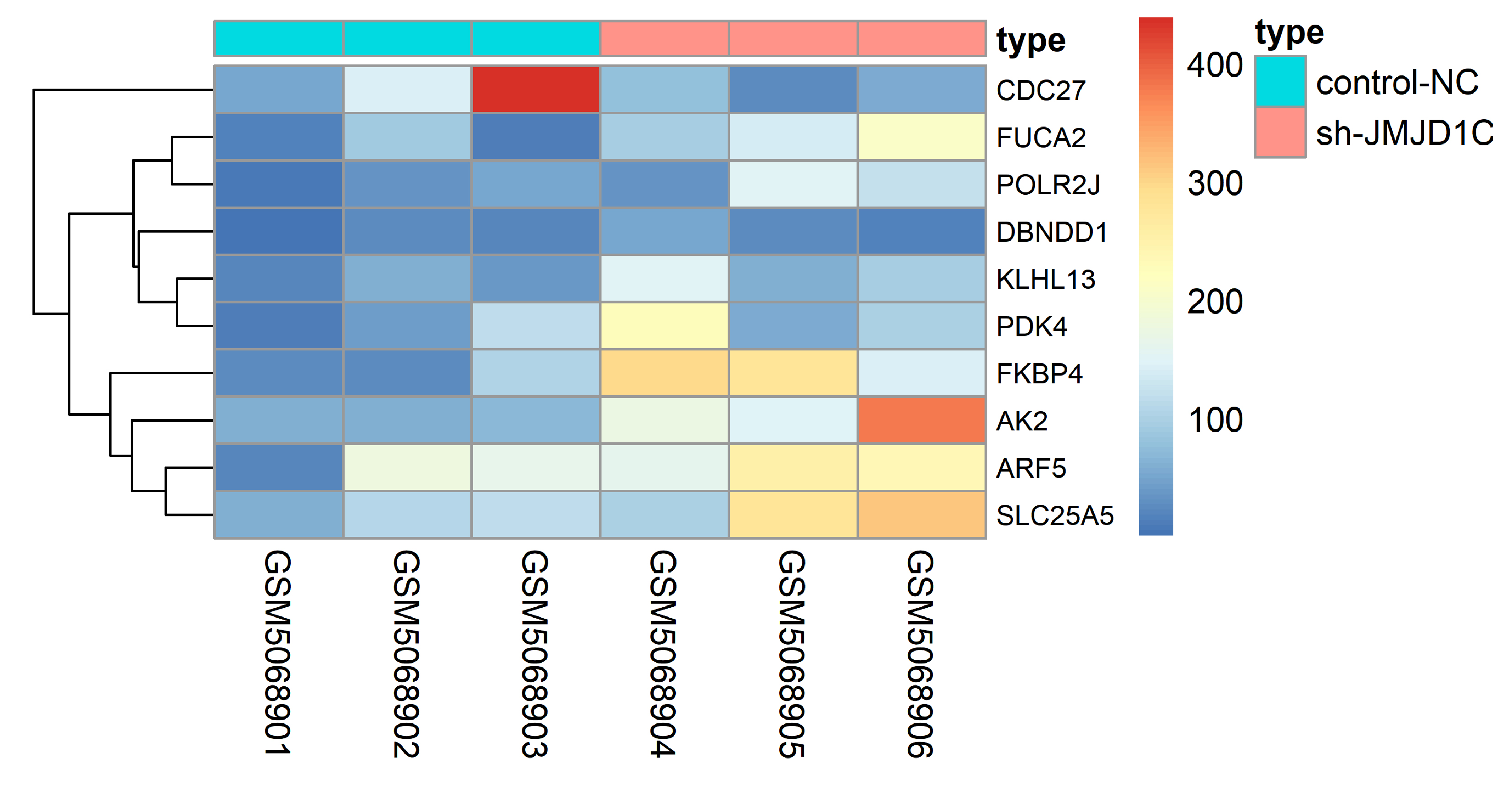

Supplement: Supplementary file 3 — SUPPLEMENTARY FIGURE 3 Heatmap of some specific JMJD1C target genes [file CTM2-11-e424-s004.jpg]
