# Supplementary material for: Histone demethylase JMJD1C promotes the polarization of M1 macrophages to prevent glioma by upregulating miR‐302a
Source: Clin Transl Med. 2021 Sep 26;11(9):e424. doi: 10.1002/ctm2.424 (PMC8473479; doi:10.1002/ctm2.424)
Supplement: Supplementary file 4 — tableS1‐S2 [file CTM2-11-e424-s003.docx]

**Supplementary Table 1** Primer sequences for RT-qPCR

| Genes | Primer sequences |
| --- | --- |
| JMJD1C (human) | F: 5'-TCCTGTCAGACCTTCCAGTGCA-3' |
|  | R: 5'-GTGGATGCAACAGACCGTAATGG-3' |
| miR-302a (human) | F: 5'-AAACGTGGATGTACTTGC-3' |
|  | R: 5'-GAACATGTCTGCGTATCTC-3' |
| miR-302a (mouse) | F: 5'-CTTAAACGTGGTTGTACTTG-3' |
|  | R: 5'-GAACATGTCTGCGTATCTC-3' |
| U6 (human) | F: 5'-GGGCAGGAAGAGGGCCTA-3' |
|  | R: 5'-GAACATGTCTGCGTATCTC-3' |
| U6 (mouse) | F: 5'-CAGCACAAAAGGAAACTCACC-3' |
|  | R: 5'-GAACATGTCTGCGTATCTC-3' |
| METTL3 (human) | F: 5'-CTATCTCCTGGCACTCGCAAGA-3' |
|  | R: 5'-GCTTGAACCGTGCAACCACATC-3' |
| SOCS2 (human) | F: 5'-GGTCGGCGGAGGAGCCATCC-3' |
|  | R: 5'-GAAAGTTCCTTCTGGTGCCTCTTTT-3' |
| GAPDH (human) | F: 5'-GTGGACCTGACCTGCCGTCT-3' |
|  | R: 5'-GGAGGAGTGGGTGTCGCTGT-3' |
| IL-1β (mouse) | F: 5'-AAGGGGACATTAGGCAGCAC-3' |
|  | R: 5'-ATGAAAGACCTCAGTGCGGG-3' |
| TNF (mouse) | F: 5'-CCTCTCATGCACCACCATCA-3' |
|  | R: 5'-GCATTGCACCTCAGGGAAGA-3' |
| CXCL9 (mouse) | F: 5'-GCAGTGTGGAGTTCGAGGAA-3' |
|  | R: 5'-AGTCCGGATCTAGGCAGGTT-3' |
| IL-6 (mouse) | F: 5'-GACTGGGGATGTCTGTAGCTC-3' |
|  | R: 5'-CACCAGCATCAGTCCCAAGA-3' |
| RETNLA (mouse) | F: 5'-CTGCTACTGGGTGTGCTTGT-3' |
|  | R: 5'-GCAGTGGTCCAGTCAACGAG-3' |
| TGFB1 (mouse) | F: 5'-GTCCAAACTAAGGCTCGCCA-3' |
|  | R: 5'-ATAGATGGCGTTGTTGCGGT-3' |
| VEGF-a (mouse) | F: 5'-GACCTCTCACCGGAAAGACC-3' |
|  | R: 5'-TCCTCTTCCTTCATGTCAGGC-3' |
| EGF (mouse) | F: 5'-TCCTCTTCCTTCATGTCAGGC-3' |
|  | R: 5'-CTGATAAGACGGACGGAGCC-3' |
| IL-23a (mouse) | F: 5'-TGGAGCAACTTCACACCTCC-3' |
|  | R: 5'-GGCAGCTATGGCCAAAAAGG-3' |
| ROS1 (mouse) | F: 5'-GGCCATCCTTTCCCAAGTGA-3' |
|  | R: 5'-GTTGACGTGGGGTGGGTAAT-3' |
| IL-12a (mouse) | F: 5'-CTCAGTTTGGCCAGGGTCAT-3' |
|  | R: 5'-TCTTCAGCAGGTTTCGGGAC-3' |
| IL-12b (mouse) | F: 5'-AGGCTGGACTGCATGATAGC-3' |
|  | R: 5'-GTAAGCAACCGACTCTCCCC-3' |
| IL-10 (mouse) | F: 5'-GCATGGCCCAGAAATCAAGG-3' |
|  | R: 5'-AATCGATGACAGCGCCTCAG-3' |
| ARG1(mouse) | F: 5'-AACCATCTGGGGCATCACAG-3' |
|  | R: 5'-ACCAGAAAGGAACTGCTGGG-3' |
| CCL22 (mouse) | F: 5'-CCCTATGGTGCCAATGTGGA-3' |
|  | R: 5'-GCAAGGCTCTTGCTGGAATG-3' |
| IL1B (human) | F: 5'-GAGCTCGCCAGTGAAATGAT-3' |
|  | R: 5'-CCTGAAGCCCTTGCTGTAGT-3' |
| TNF (human) | F: 5'-AGAACTCACTGGGGCCTACA-3' |
|  | R: 5'-GCTCCGTGTCTCAAGGAAGT-3' |
| CXCL9 (human) | F: 5'-GATTGGTGCCCAGTTAGCCT-3' |
|  | R: 5'-CCACCGGACAGCACTCTAAA-3' |
| IL6 (human) | F: 5'-TCTCAACCCCCAATAAATATAGGAC-3' |
|  | R: 5'-GATGCCGTCGAGGATGTACC-3' |
| IL10 (human) | F: 5'-TTCCAGTGTCTCGGAGGGAT-3' |
|  | R: 5'-GCTGGCCACAGCTTTCAAGA-3' |
| ARG1 (human) | F: 5'-GTCTGTGGGAAAAGCAAGCG-3' |
|  | R: 5'-CACCAGGCTGATTCTTCCGT-3' |
| CCL22 (human) | F: 5'-CCTACTCTGATGACCGTGGC-3' |
|  | R: 5'-GAGAGTTGGCACAGGCTTCT-3' |
| IL4 (human) | F: 5'-ATGGGTCTCACCTCCCAACT-3' |
|  | R: 5'-TCTGTTACGGTCAACTCGGTG-3' |

Notes: JMJD1C, jumonji domain containing 1C; METTL3, methyltransferase like 3; SOCS2, suppressor of cytokine signaling 2; GAPDH, glyceraldehyde-3-phosphate dehydrogenase; IL, interlukine; TNF, tumor necrosis factor; CXCL9, C-X-C motif chemokine ligand 9; VEGF, vascular endothelial growth factor; ROS, reactive oxygen species; ARG, arginase; F, forward; R, reverse.

**Supplementary Table 2** Primer sequences used in ChIP-PCR

| Genes | Primer sequences |
| --- | --- |
| JMJD1C | F: 5'-TCTTGTGGTAATGGTTTTAGCTG-3' |
|  | R: 5'-GGTGGGTCTGGATCCTTTTT-3' |

Notes: JMJD1C, jumonji domain containing 1C; F, forward; R, reverse.
